# Advancing quantitative techniques to improve understanding of the skeletal structure-function relationship

**DOI:** 10.1186/s12984-018-0368-9

**Published:** 2018-03-20

**Authors:** Frances T. Sheehan, Elizabeth L. Brainerd, Karen L. Troy, Sandra J. Shefelbine, Janet L. Ronsky

**Affiliations:** 1 National Institutes of Health, Bethesda, MD USA; 2 Brown University, Providence, RI USA; 3 Worcester Polytechnic Institute, Worcester, MA USA; 4 Northeastern University, Boston, MA USA; 5 University of Calgary, Calgary, AB Canada

## Abstract

Although all functional movement arises from the interplay between the neurological, skeletal, and muscular systems, it is the skeletal system that forms the basic framework for functional movement. Central to understanding human neuromuscular development, along with the genesis of musculoskeletal pathologies, is quantifying how the human skeletal system adapts and mal-adapts to its mechanical environment. Advancing this understanding is hampered by an inability to directly and non-invasively measure in vivo strains, stresses, and forces on bone. Thus, we traditionally have turned to animal models to garner such information. These models enable direct in vivo measures that are not available for human subjects, providing information in regards to both skeletal adaptation and the interplay between the skeletal and muscular systems. Recently, there has been an explosion of new imaging and modeling techniques providing non-invasive, in vivo measures and estimates of skeletal form and function that have long been missing. Combining multiple modalities and techniques has proven to be one of our most valuable resources in enhancing our understanding of the form-function relationship of the human skeletal, muscular, and neurological systems. Thus, to continue advancing our knowledge of the structural-functional relationship, validation of current tools is needed, while development is required to limit the deficiencies in these tools and develop new ones.

## Background

Motion in humans and other animals (e.g., walking, running, leaping, flying, and swimming) emerges from the interaction of skeletal shape, strength, and stiffness; musculotendon architecture and mechanics; and neural control. Yet, it is the skeletal system that creates the overall framework for functional movement. The overall shape of bones converts muscle shortening into gross motion; and the specific shape of articular surfaces both guides and constrains motion. Neuromechanical systems, sometimes with surprising self-stabilizing properties, emerge from the interaction of this mechanical complexity with the intricacies of motor and sensory control [1]. Thus, quantifying how the skeletal system adapts and mal-adapts to these stresses is central to understanding neuromuscular development, along with the genesis of musculoskeletal pathologies.

The internal and external geometry of bones and their articular surfaces are the scaffold on which motion is built. Bone geometry varies among individuals and changes substantially during development. It is also influenced by physical activity, injury, and pathology. This geometry is also influenced by evolution. Among the more than 60,000 species of vertebrate animals, skeletal shape varies widely with evolutionary adaptation, such as bat and bird wings for flight and long frog legs for jumping. Discovering robust form-function relationships of the skeletal system in both human and non-human animals is required to advance our understanding of skeletal remodeling, musculoskeletal pathologies, and musculoskeletal function.

New imaging and modeling methods have the potential to advance our knowledge of form-function relationships rapidly. Studies evaluating and models including detailed musculoskeletal morphology, be it patient-specific or species-specific, are an important frontier in the field of biomechanics and neural control of movement. Thus, we need to exploit the tools we currently have at our disposal and continue to develop and validate new ones for the accurate and precise quantification of in vivo musculoskeletal properties and the functional movement they enable. The focus of this review is to provide an overview of techniques for advancing understanding of this relationship with specific examples of adaptation and mal-adaptation within the developing skeleton, mature skeleton, musculoskeletal interactions at the joint level, and vertebrates.

## The developing skeleton

It has been known for centuries that altering the load on the bone affects bone development and growth. In ancient China, small feet were a sign of beauty and girls’ feet were bound to prevent growth [2]. In Indian and African tribes, the heads of children were bound between planks to elongate the skull, which was considered a sign of nobility [3]. Modern research has confirmed that high loads induce bone formation and reduced loads result in bone resorption, as proposed by Julius Wolff in the 1890s [4]. Growing bone is particularly sensitive to its loading environment because the ossification process can be altered with mechanical loads. This “mechano-sensitivity” allows deformities and dysplasias to develop rapidly when exposed to inappropriate loading. However, it also offers great potential for clinical interventions that obtain appropriate bone loading to ensure normal growth.

Time-varying multi-directional bone loading, resulting from both muscle and external forces, in concert with biochemical factors (e.g., hormones, growth factors, nutrients [5]) directly regulates bone growth. Although the loading environment is complex, it has three key elements: 1) number of loading cycles; 2) magnitude of loading; and 3) direction of loading. The number of loading cycles is the simplest to measure. The direction and magnitude of bone loading are more challenging to assess and typically rely on analysis of motion patterns. Combining motion analysis with musculoskeletal models using inverse kinematics [6], optimization algorithms [7], multi-objective optimization functions [8], or computed-muscle control methods [9] provides estimations of muscle forces, as well as the forces and moments applied to the skeleton. It is these loads that determine the mechanical environment of the growing bone. When musculoskeletal modeling is combined with finite element (FE) bone modeling, tissue-level stresses can be determined. These tissue level stresses, in turn, can be used to predict ossification patterns and ultimately changes to the overall bone shape, such as in developmental dysplasia of the hip [10, 11] or bone deformities in cerebral palsy [12, 13].

As insightful as these combined motion analysis and modeling approaches have been, understanding the initial skeletal shape is critical to obtaining reliable estimates of joint loads and tissue stresses, particularly when evaluating the pediatric population. This problem becomes even more complicated in the presence of pathology when both bone size and shape can be drastically altered from the typical adult model [14, 15]. Studies have shown that subject-specific musculoskeletal models estimate significantly reduced joint forces and moments, compared to using scaled generic models [16–18]. For example, estimated joint moments during gait in children with cerebral palsy are reduced when the generic model is replaced with subject-specific morphology [18]. This indicates that the gait in children with cerebral palsy may be “optimized” for the bone shape; or alternatively, the bone shape may be “optimized” for the gait. Likewise, FE models with subject specific growth plate shapes have different tissue stresses than generic growth plate shapes [19]. Thus, taking into account specific geometry is critical in understanding mechanical function.

Another challenge is accurately modeling neuromuscular control parameters for children with altered gait patterns. Currently, musculoskeletal models can predict quite accurately muscle firing patterns and joint loads during typical walking. Yet, children with disabilities (e.g., cerebral palsy, spina bifada) presumably do not use the same optimization criteria in their gait. Mathematically capturing the spasticity, affected neuro-motor control, and muscle tone of these children is challenging. However, these are the populations where altered gait affects bone formation and where the prediction of bone growth is of great clinical interest. Although limitations still remain in measuring or even estimating the loading environment experienced by the pediatric skeletal system, studies combining musculoskeletal and FE modeling have shown that altered loading affects the progression of the growth front and could result in bone deformities [13]. Thus, as our measurement and modeling tools improve, we will have an increased capacity to predict interventional outcomes, streamlining our ability to generate successful patient-specific treatment plans.

While the relationship between form and function is exaggerated in the growing skeleton due to rapid growth, the currents tools have been designed to assess normal adult function. These tools are not adequate for a growing child with bone deformities, yet this is where the clinical need for assessment is highest. Thus, a focused effort is needed to adapt current experimental and modeling techniques to the study of pediatric populations.

## The mature skeleton

After skeletal maturity, bone maintains its capacity to adapt to its mechanical loading environment. This adaptation is driven primarily by strain. Specifically, for a given applied force, weak bones experience larger strains, whereas strong bones experience lower strains. This elicits increased adaptation in the weaker bone, eventually resulting in stronger bone – a phenomenon described by some as a “mechanostat” [20], with bone having a mechanical set point, similar to a thermostat. Although the actual process is understood to be more complex than the analogy implies, due to other physiologic factors that influence bone adaptation, the basic principle has been upheld through both retrospective and prospective observation [21–24]. For example, bone adaptation in skeletally mature women has been observed to be site specific and related to energy equivalent strain, with high strain regions experiencing more bone apposition than low strain regions [25].

Although the relationship between mechanical signals and bone adaptation has been extensively studied in animals [26], this relationship is not well understood in humans due to difficulties in noninvasively measuring both the stimulus and the change in bone structure. Thus, mechanical loading in humans is frequently estimated through either retrospective physical activity surveys [27–29] or more directly via measures based on ground reaction force or body segment accelerations [30].These measures are limited in that they do not consider how bone structure, which is highly variable even among healthy individuals, affects bone strain – the driver of bone adaptation. Bone mineral density (BMD) is widely used as a surrogate measure of bone strength. It is inversely related to strain for a given force, but only explains about 50% of the variance in the relationship [31]. Furthermore, bone strain is highly variable, while BMD is not. For example, in a sample of 23 women with fairly homogenous characteristics, the same simulated external force (300 N) on the distal radius results in a 6-fold variation in mean strains, but only a 2-fold variation in BMD [32]. Collectively, this indicates that the underlying bone structure is just as important as the density of bone in determining how much strain it will experience for a given force. And, since bone adaptation is strain-driven, it may also partially explain why exercise-based therapies aimed at increasing BMD work for some people, but not others, as exercises are generally prescribed based on force, not bone strain.

In the past decade, three-dimensional imaging methods such as CT and MR imaging have become useful for noninvasively quantifying bone structure, from the micro to the macro scale [33–37]. On the macro-scale, patient-specific CT-based FE models have been shown to accurately estimate bone strain [32, 38, 39] for a variety of physiologic loading scenarios. Not only does CT data provide the basis for patient-specific geometry, these data can account for variations in BMD that are detectable with x-ray, explaining approximately 85% of the variance in surface strain. The primary limitation to these models is that generally they are validated only in specific loading scenarios and include assumptions and simplifications that limit their application. Furthermore, creating patient-specific FE models is labor-intensive. On both the micro- and macroscale, CT data can be analyzed quantitatively to calculate parameters such as bone mineral content and BMD, which have been related to bone tissue elastic modulus [40–43]. High resolution peripheral quantitative CT (HR-pQCT) has rapidly become a method of choice for noninvasive measurement of bone microstructure in living humans. With relatively low radiation exposure (around 3 micro-Sieverts per scan, or roughly half a day’s background exposure), HR-pQCT and FE models based on HR-pQCT data have provided useful information regarding specific microstructural changes associated with bone fragility [44]. However, HRpQCT is currently expensive, limited to imaging the extremities, and, practically, it cannot acquire data on more than one or two centimeters of a limb, due to the long scan time and large file sizes associated with very high resolution data.

Bone structure and physical activity are intimately linked, with healthy bones facilitating an active lifestyle and an active lifestyle contributing to healthy bones. The achievement of high peak bone strength during young adulthood imparts lifelong protection against fragility fractures [45]. Subject-specific FE models and microstructural measurements are providing useful insights into how mechanical loads influence bone structure, and how bone structure affects the resulting strain. There is a need for high quality, prospective data linking specific characteristics of mechanical signals and physiologic traits to bone adaptation in healthy and clinical populations. Many challenges exist, such as identifying the threshold between optimal and damaging mechanical loading on both hard and soft tissue, exploiting the natural feedback system to safely strengthen bone in vulnerable populations, and identifying characteristics of people who might respond to such interventions a priori.

## Mal-adaptation at the joint level

As joints enable articulation, healthy musculoskeletal function involves the joint tissues’ (e.g., bone, cartilage, tendon, ligaments, etc) ability to continuously adapt their structure and biology to their mechanical loading environment. The specific factors and conditions required to maintain the homeostasis for healthy joint tissues remain poorly understood. Developing techniques to study conditions that result in joint degeneration are shedding light on what is required to maintain healthy joint homeostasis [46–54].

One example of joint degeneration is tibiofemoral osteoarthritis (OA), which severely impacts a patient’s quality of life. Treatments to date are limited, with the primary option being pain and inflammation management and, eventually, joint replacement. Many factors, such as trauma, can initiate OA. For example, 12 to 20 year follow ups of athletes who sustained anterior cruciate ligament (ACL) ruptures reveal that 40–50% of these athletes have OA by ages 25–54 years. Altered joint kinematics and kinetics, associated with ACL deficiency (ACLD) [49, 55–59], are theorized to cause a shift in tibiofemoral cartilage contact locations and alter cartilage loading patterns [60]. Such an abrupt injury-induced change in the joint loading environment may increase cartilage susceptibility to damage at regions ill-adapted to withstand these altered loads [61–64]. Over years, this interaction causes tissue damage and loss, leading to clinically symptomatic OA. To date, this proposed framework has been difficult to verify directly in humans due to limits in our experimental measurement tools.

Various highly accurate imaging tools can individually provide data in regards to OA, but the true power comes from leveraging these tools off each other. For example, biplanar video-fluoroscopy (BVF) systems, which combine fluoroscopic imaging with magnetic resonance or CT images and use novel calibration approaches, provide the opportunity to obtain submillimeter accuracies in quantifying in vivo 3D bone movements [65–68]. This level of accuracy can likely detect differences between healthy and individuals with ACLD or OA. In addition, during early OA, cartilage exhibits swelling and softening, which can lead to cartilage degeneration [69, 70]. Magnetic resonance imaging of cartilage based on T2 relaxometry has been used to study these early structural and compositional changes, as the sequence is sensitive to collagen architecture, proteoglycan, and water content [71, 72]. Specifically, higher T2 values have been reported in the tibiofemoral cartilage of individuals with OA and subjects with ACLD [73, 74]. Finally, high resolution magnetic resonance images can provide 3D structural images for deriving bone and cartilage models, along with FE models of cartilage. Combining these tools enables the investigation of in vivo cartilage deformation associated with the bone movement. Quantifying the corresponding T2 values within contact and non-contact regions for the tibiofemoral cartilage provides information regarding cartilage composition within these regions. Lastly, incorporating measures of muscle activations allows insights into changes in neuro-muscular control associated with the various conditions.

A recent pilot study using this integrated imaging approach, with four healthy and four ACLD individuals, found a substantially higher loading rate under static loading for the ACLD individuals in comparison to the healthy controls [51, 52]. Altered regions of tibiofemoral cartilage contact, as well as altered T2 values in contact and non-contact regions were identified during walking. The average T2 values for the ACLD limbs were higher than those of the ACL intact limbs, which could be potentially associated with early OA. Thus, applying a combination of tools has provided preliminary evidence on the link between injury, change in cartilage loading, and OA.

Characterizing the relationship between cartilage structure and composition (T2 values) with dynamic loading will likely provide cartilage mechanical function information for early OA detection. The key components involve identifying changes in joint: kinematics and kinetics, morphology, cartilage structure, and neuromuscular control.This integrated structure-function approach provides promise for advancing the understanding of mechanisms of cartilage homeostasis, as well as mal-adaptations, such as cartilage degeneration leading to OA. Further development in 3D-2D registration approaches, numerical simulations and integration of techniques are required to enable these functional dynamic imaging approaches to advance understanding of the in-vivo mechanics of a larger spectrum of healthy joints and those afflicted with pathology.

## Skeletal shape and motion across the vertebrates

As with studies on humans, studies of skeletal shape and motion in non-human animals contribute to our understanding of 3D joint function, to the development of biologically-inspired devices, and to understanding the natural world. Model animal species, such as mice and rats, are developed specifically for biomedical research and benefit from uniformity. At the other extreme, comparative studies of a wide range of species can yield insights beyond what can be learned from direct study of humans and even other mammals. The more than 60,000 species of extant vertebrates exhibit a wide variety of articular surface shapes that guide the motion of joints, permitting some motions and constraining others. But to develop general principles for how articular shape relates to motion, it is necessary to measure both shape and motion simultaneously.

The shape of articular surfaces can be determined with CT or magnetic resonance imaging and combined with bone motion from BVF to visualize bone shape and motion simultaneously. This combination of techniques has now been applied extensively to study joint function in both humans and other animals [65–67, 75–78]. In most non-human animals, the registration of 3D bone models to 2D bi-planar video fluoroscopy images can be facilitated by surgical implantation of small (< 1 mm) radiopaque beads into the bones [79]. Combining this motion with a CT scan of the same individual animal yields a precise and accurate (within ±0.1 mm) XROMM animation of bone shape and motion [75, 76]. In most studies of human joints, marker beads cannot be implanted into the bones, so the 3D to 2D registration must be done by markerless matching of bone shape data to fluoroscopy images [65–67, 77, 78]. With or without markers, and in humans or other animals, these techniques are yielding unprecedented insights into the relationship between skeletal shape and motion.

For example, using XROMM to study the biomechanics of breathing in lizards is yielding general insights into lung ventilation in all tetrapods, including humans [80], as well as specific insights into the similarities, differences, and evolution of ribs and intercostal muscles in various vertebrate groups [81]. Relative to mammals, rib motions for breathing in lizards are exaggerated because lizards lack a diaphragm muscle to assist with lung expansion. Lizards rely entirely on their rib motions for breathing, making them good subjects for deriving general principles for how rib shape and motion interact to expand the thorax [80].

Historical studies on the skeletal form-function relationship in non-human animal models has greatly advanced our understanding of this relationship in humans. A challenge going forward will be to develop the data management tools needed to synthesize skeletal shape and motion data from multiple studies and species. The 3D animations resulting from combining CT or MR imaging data with bi-planar video fluoroscopy are data rich and could be re-analyzed with new methods to answer new questions. But data must be managed and shared in a standardized manner to facilitate sharing and reuse, and this is particularly true for comparative studies among species [82]. Toward developing general principles for how articular shape relates to motion, comparative studies of diverse joints from many species will be necessary for developing statistical models of joint function [83].

## Conclusion

Although all functional movement is born out of the interplay between the neurological, skeletal, and muscular systems, it is the skeletal system that forms the basic framework from which functional movement is created. Not only does it provide the structure to which muscles attach, its shape and size affect the overall ability for a muscle to generate torque, and the skeletal system creates a parameter space for movement. Thus, central to understanding human neuromuscular development, along with the genesis of musculoskeletal pathologies, is an understanding of how the human skeletal system adapts and mal-adapts to its mechanical environment. Advancing this understanding has been hampered by an inability to directly measure the in vivo strains, stresses, and forces on bone, non-invasively in humans. Thus, we have turned to the animal model to evaluate how the skeletal system adapts to long-term stresses through evolution. The animal model also enables direct in vivo measures that are not available in human subjects, providing information in regards to both skeletal adaptation and the interplay between the skeletal and muscular systems. Recently, there has been an explosion of new imaging and modeling techniques that are beginning to provide the in vivo measures of human skeletal form and function that have long been missing. Combining multiple modalities (e.g., a BVF with magnetic resonance image based models, gait analysis with dynamic modeling, CT models with FE models, etc.) has proven to be one of our most valuable resources in enhancing our understanding of the form-function relationship of the human skeletal system, along with the muscular and neurological systems. Many challenges exist in our efforts to fully understand the form-function relationship of the skeletal system, yet progress in the development and validation of tools to measure or estimate skeletal properties has paid high dividends, not only in terms of increased general knowledge, but in the prevention and treatment of various debilitating musculoskeletal impairments and pathologies.

## Abbreviations

ACL: Anterior cruciate ligament
ACLD: ACL deficiency
BVF: Biplanar video-fluoroscopy
FE: Finite element
HRpQCT: High resolution peripheral quantitative CT
MR: Magnetic resonance
OA: Osteoarthritis
PF: Proteoglycan
PTOA: Post-traumatic osteoarthritis
TF: Tibiofemoral
XROMM: X-ray reconstruction of moving morphology
